# Immobilization of Pepsin on Different Nanomaterials and Its Application in Obtaining Bioactive Peptides

**DOI:** 10.1002/jssc.70546

**Published:** 2026-10-10

**Authors:** Annie N. Alves, Márjorie C. P. Porfirio, Priscilla A. Nascimento, Jéssica F. Borges, Wenrong Yang, Motilal Mathesh, Paulo Bonomo, Rafael da C. I. Fontan, Renata C. F. Bonomo

**Affiliations:** ^1^ Process Engineering Laboratory State University of Southwestern of Bahia (UESB) Itapetinga Brazil; ^2^ School of Life and Environmental Sciences Deakin University Geelong Australia

**Keywords:** biocatalyst, enzyme conformation, immobilization, nanomaterials

## Abstract

Enzyme immobilization is frequently used in catalytic processes as an alternative to improve enzyme activity and stability. Among the materials used as a support, nanomaterials have been successfully reported in the literature, mainly due to its high specific area. In this study, the nanomaterials graphene, reduced graphene oxide, boron nitride nanosheets, and gold nanoparticles were evaluated as a support for pepsin immobilization. The effectiveness of this process was evaluated by the content of immobilized protein and activity of immobilized pepsin, determined through casein hydrolysis, as well as circular dichroism to verify possible alterations in enzyme structure. The antioxidant properties of hydrolysates were assessed by DPPH, ABTS, FRAP, and β‐carotene. All the materials studied presented a high efficiency of immobilization, with over 80% of the pepsin content bound to the support, the use of graphene derivatives as support led to a reduction in proteolytic activity, with the immobilized pepsin retaining only 6% of its activity. The 45 nm gold nanoparticle led to an increase in proteolytic activity from 0.050 to 0.104 U. No effect of amino group functionalization of boron nitride nanosheet was observed. The variations determined in the secondary structure, especially in the β‐sheet content, corroborate the variations in activity after immobilization. The change in casein hydrolysates using pepsin immobilized in nanomaterials can be verified by reversed‐phase chromatography. This change in the enzyme conformation affects the antioxidant properties of the casein hydrolysates. The results found in the present study contribute to the development of new biocatalysts based on the immobilization of enzymes in nanomaterials.

## Graphical abstract

A study on the immobilization of pepsin on various nanomaterials (graphene oxide, boron nitride, and gold nanoparticles) was conducted. The immobilized pepsin was used to generate bioactive peptides, which were then evaluated for their antioxidant properties.

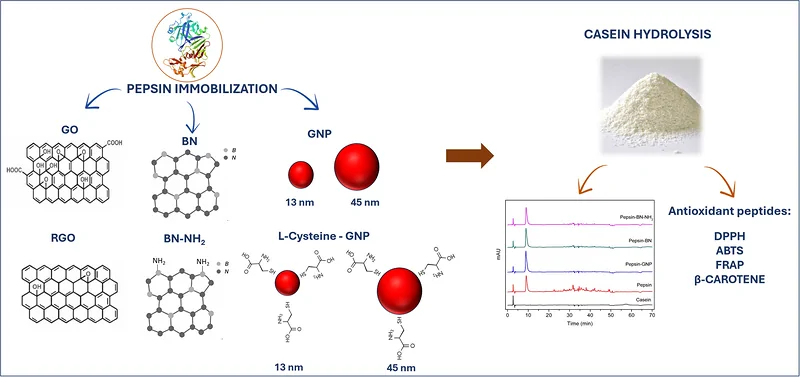

## 1 Introduction

Pepsin, extracted from gastrointestinal juice, belongs to the group of aspartic proteases since it has two aspartic acid residues in its active site. Like enzymes belonging to this group, it has better activity in acidic conditions, at a pH of around 2.0–4.0 [1]. Pepsin generally acts preferentially on the peptide bond containing phenylalanine, leucine, and methionine. Its action is also favored by aromatic amino acids such as tyrosine, tryptophan, and phenylalanine [2].

Their application in the production of nutraceuticals is gaining prominence due to their ability to release bioactive peptides after protein hydrolysis. In vivo and in vitro studies have demonstrated the capacity of these peptides to contribute to human health by acting directly on the reduction and treatment of cardiovascular diseases, as well as by exerting immunomodulatory and antimicrobial functions. Peptides with antioxidant properties have attracted industry interest because they can inhibit the formation and propagation of free radicals, thereby preventing pro‐inflammatory processes in the human body [3, 4, 5, 6].

Despite their great relevance, the industrial application of enzymes has some limitations, such as low stability in solution and, in the case of proteases, the autohydrolysis process must also be considered [7, 8].

In this context, enzyme immobilization emerges as a promising strategy, as it can enhance the catalytic and structural stability of enzymes, improve their thermostability, and allow for the reuse of the biocatalyst [8]. For this approach to be effective, the appropriate choice of immobilization method and support material is essential.

Different types of materials have been applied as supports for protease immobilization. Santos et al. [9] and Souza Junior et al. [10] evaluated the activated carbon obtained from agro‐industrial waste for and pepsin immobilization. Li et al. [11] evaluated the interaction of the proteases as pepsin and trypsin with gold nanoparticles. Mehdi et al. [12] immobilized proteases in a new material based on chitin and starch. Covalently functionalized silica was used for pepsin immobilization and applied for collagen extraction [13].

In addition to these materials, the use of nanomaterials as a support for immobilization has been highlighted. Nanomaterials are materials with at least one dimension in the nanometer scale, ranging from 1 to 100 nm [14]. The use of these materials as supports for immobilization, enzymatic modulation, and biosensors is widely reported [15, 16]. Nanomaterials offer several advantages due to their high surface area‐to‐volume ratio and easiness of characterization by quantum effects. These characteristics enable efficient enzyme binding to the support with relatively low resistance to mass transfer, facilitating easier access of the enzyme to the substrate. The easy dispersion of these materials in an aqueous solution facilitates characterization studies [17, 18]. Materials used to produce nanocatalysts include graphene oxide and its derivatives [18, 19, 20], boron nitride [21], and gold nanoparticles [17, 22, 23]. Different types of enzymes can bind to these supports by adsorption, covalent bonds, ionic interactions, or affinity. These materials are attractive due to their relatively simple synthesis on a laboratory scale and their biocompatibility.

Graphene oxide (GO) is a two‐dimensional material formed by carbon atoms arranged in a hexagonal lattice obtained from the exfoliation of graphite. The main characteristic that makes it so widely used in the production of biomaterials is the presence of oxygenated functional groups with hydroxyl (‐OH), epoxy (‐O‐), and carboxyl (‐COOH) on its surface, allowing the binding of proteins without the need for functionalization with functional groups. In addition, the presence of these groups makes graphene oxide hydrophilic, thus allowing its dispersion in aqueous media and polar solvents, facilitating its application. Chemically reduced graphene oxide (RGO) is a derivative of GO synthesized using L‐ascorbic acid as a reducing agent. The successful use of GO as a support for immobilization is reported in the literature [19, 20, 24].

Unlike GO, RGO has fewer oxygenated groups on its surface, making it more hydrophobic [18]. Boron nitride (BN) nanosheets are two‐dimensional materials. Like GO, their atoms are arranged in a hexagonal configuration, also known as “white graphene oxide.” However, unlike GO, BN is formed by alternating nitrogen and boron atoms [25, 26].

Gold nanoparticles (GNP) are synthesized using chloroauric acid (HAuCl_4_) as the gold precursor and sodium citrate as the reducing agent. The size of the nanoparticles can vary from 8 to 100 nm and can be controlled by the concentration of the reducing agent used for the synthesis. The high surface‐to‐volume ratio of GNP particles allows for a vast immobilization capacity of this material [17, 27]. In addition to adsorption, biomolecules can be immobilized by binding functional groups to the GNP surface [28].

Given the potential applications in biological systems, researchers have focused on protein–nanoparticle complexes to understand their interactions and the conformational changes of proteins. Therefore, this study aimed to evaluate the use of nanomaterials such as graphene oxide, boron nitride nanosheets, and gold nanoparticles in the immobilization of pepsin and to investigate the use of these biocatalysts in obtaining bovine casein protein hydrolysates with antioxidant activity.

## 2 Materials and Methods

### 2.1 Materials

Pepsin (CAS: 9001‐75‐6), tyrosine purity 98% (CAS:60‐18‐4), casein (CAS: 9000‐71‐9), bovine serum albumin (BSA) (CAS: 232‐936‐2), bovine serum casein (CAS: 9000‐71‐9), L‐cysteine (CAS:52‐90‐4), HAuCl_4_ (CAS 16903‐35‐8), and graphite (7782‐42‐5) were purchased from Sigma Aldrich AUS. Bradford protein determination solution (CAS 23200) was purchased from Thermo Fisher‐AUS for determining protein content. The remaining reagents were of analytical grade PA‐AS.

### 2.2 Nanomaterial Synthesis

#### 2.2.1 Carbon‐Based Material

The synthesis of graphene oxide was carried out as proposed by Matesh et al. [18]. Note that 2.0 g of graphene flakes were mixed with sulfuric acid (15 mL) and sonicated for 6 h. The obtained material was dried at 70°C in an oven. Sulfuric acid (120 mL) and potassium permanganate (15 g) were added to the pre‐oxidized graphite, and the mixture was stirred in an ice bath for 2 h. The solution was then diluted with water (250 mL) and stirred for 2 h in an ice bath. After adding 700 mL of water, 20 mL of hydrogen peroxide was quickly added to the mixture and left to rest for 12 h. The sediment was separated into 50‐mL Eppendorf tubes and spun (Eppendorf Centrifuge Model 5810 R, Australia) at 15 652 *g* for 30 min. For washing, the pellets were mixed with a 1:10 hydrochloric acid solution and centrifuged at 15 652 g for 10 min, then they were rinsed with distilled water at 15 652 g for 10 min to remove the acid. Washing was repeated until a yellow color appeared in the supernatant.

The chemically RGO was produced according to Mathesh et al. [18]. Note that 100 mg of L‐ascorbic acid was added to 10 mL of GO solution, which was previously kept in an ultrasonic bath for 15 min and then stirred for 2 h. The solution was washed with distilled water and centrifuged at 15 652 g for 15 min (Eppendorf Centrifuge Model 5810 R, Australia). Then, the pH of the mixture was adjusted to 7.0 with sodium phosphate buffer (0.01 M, pH 7.0).

### 2.3 Boron Nitride Nanosheets and NH_2_ Functionalized BN

The BN and NH_2_ functionalized BN nanoparticles (BN‐NH_2_) used in these studies were provided by Deakin University in Australia, where part of the work was conducted. The synthesis of boron nitride nanosheets was carried out through the mechanical exfoliation of hexagonal boron nitride crystals, using a four‐station planetary ball mill system [21]. This process breaks the B‐N bonds and causes damage in the structure, particularly at the edges, increasing the reactivity and facilitating the functionalization through hydroxyl or amino groups [21].

### 2.4 Gold Nanoparticle

GNPs were synthesized as reported by Zhang et al. [29]. Note that 1.0 mL of HAuCl_4_ solution (5.0 × 10^−3^ M) was added to 18 mL of boiling water, followed by 0.5 mL of sodium citrate tribasic dihydrate (0.5% w/w). For synthesizing 13 and 45 nm GNPs, 1.0 mL and 0.365 mL of sodium citrate solution were used, respectively. After changing the color to red, the mixture remained heated for about 10 min. The solution was cooled to room temperature, and the final volume was adjusted to 20 mL. The concentration of gold nanoparticles was approximately 2.5 × 10^−4^ M. For the synthesis of L‐cysteine‐functionalized gold nanoparticles (GNP/Cys), 0.1 mL of 1.0 × 10^−4^ M L‐cysteine solution in sodium citrate‐citric acid buffer (0.01 M pH 5.5) was added to 5.0 mL of GNP solution and kept under stirring for 2 h, and then centrifuged (24 149 g, 30 min) to remove excess unbound amino acid. After centrifugation, the supernatant was removed, and the pellet was solubilized in distilled water in an ultrasonic bath.

### 2.5 Pepsin Immobilization on Nanomaterials

#### 2.5.1 Pepsin Immobilization on Carbon‐Based Nanomaterials

For pepsin immobilization on GO (Pepsin‐GO), the effect of enzyme concentrations of 2.5, 5.0, 7.5, and 10 mg/mL in 0.05 M glycine‐HCl buffer, pH 3.0, was evaluated. Note that 0.5 mL of enzyme solution was added to 1.5 mL of GO solution. The mixture was stirred for 1 h in an ice bath to avoid changes in the enzyme structure. After immobilization, the samples were washed with the buffer and centrifuged to remove the supernatant, which was later used for protein quantification. This process was repeated three times.

After the initial tests, pepsin immobilization was conducted using a 0.0035 mg/mL concentration. At this stage, the effect of the medium's hydrophobicity was evaluated using RGO (Pepsin‐RGO).

#### 2.5.2 Pepsin Immobilization on Boron Nitride

The immobilization of pepsin (0.5 mL) on BN (Pepsin‐BN) and BN (1.5 mL) functionalized with an amine group (BN‐NH_2_) (Pepsin‐BN‐NH_2_) was conducted at pH 3.0, using 0.05 M glycine‐HCl buffer and an enzyme concentration of 0.0035 mg/mL. A total of 1.5 mL of support solution (2 mg/mL) and 0.5 mL of enzyme solution were used. The immobilization procedure and washing steps were performed as described previously. As previously mentioned, the immobilization assay was conducted in an ice bath, and successive washes were done to remove the protein content that was not immobilized.

#### 2.5.3 Pepsin Immobilization on Gold Nanoparticles

Pepsin immobilization on GNP (Pepsin‐GNP) and GNP/Cys (Pepsin‐GNP/Cys) was performed by adding 0.1 mL of enzyme solution containing 0.035 mg/mL pepsin (0.05 M glycine‐HCl buffer, pH 5.0) to 0.9 mL of the GNP solution. In preliminary tests performed at a more acidic pH, significant aggregation of the GNP/Cys occurred, compromising both the stability of the material and the reproducibility of subsequent pepsin immobilization experiments. After stirring, the mixture was refrigerated for 12 h and then centrifuged to remove unbound pepsin, followed by successive washes with the immobilization buffer.

### 2.6 Determination of Protein Content

The protein content was determined according to the Bradford reagent manufacturer's instructions. Note that 1.5 mL of the reagent was added to 30 µL of the sample and incubated for 5 min at room temperature, and absorbance at 595 nm was measured. Bovine serum albumin was used as a standard for constructing the calibration curves. The adsorption capacity was evaluated based on the difference between the initial protein concentration added to the process and the amount remaining after the immobilization period.

### 2.7 Determination of Enzymatic Activity

The enzymatic activity was performed from the hydrolysis of bovine casein. An aliquot of enzymatic solution (0.1 mL) was added to 1.0 mL of 1.0% casein solution at pH 3.0 (0.05 M glycine‐HCl). The reaction was carried out at 37°C for 10 min, and trichloroacetic acid was added, followed by centrifugation (2683 *g*, 10 min). Note that 1.0 mL of the supernatant was added to 5.0 mL of sodium carbonate (500 mM), followed by 1.0 mL of Folin‐Ciocalteu solution (20%). The mixture was incubated for 30 min at 40°C, and the absorbance at 660 nm was determined (Agilent Spectrophotometer‐Model Cary 300 UV‐VIS). One unit of enzyme activity was defined as the amount of enzyme required to produce 1 µmol of tyrosine per minute. The activity of the immobilized enzyme was determined using a similar procedure, using 1.0 mL of the resuspended pellet added to the casein solution. The following steps were conducted, as cited above. The efficiency of the immobilization process was evaluated based on the relative activity of the biocatalysts produced in comparison to the enzymatic activity of the free enzyme.

### 2.8 Characterization of Biocatalysts

The UV‐VIS spectroscopy characterized the free and immobilized form enzymes (Agilent Cary 300). The spectra were obtained in continuous mode from 800 to 200 nm using a quartz cuvette at a scan rate of 500 nm/min and a data collection interval of 1 nm.

The secondary structure of free and immobilized pepsin was characterized by circular dichroism (CD) (CD Jasco J‐815, NSW‐ Australia). The spectrum was collected from 260 to 190 nm with a 1 mm quartz cuvette at room temperature and in a nitrogen atmosphere, with a scan rate of 50 nm/min and a bandwidth of 1 nm. The CD spectra represent the average of three consecutive scans obtained by the instrument. The secondary structure of pepsin was estimated using the BESTSEL online server [30].

The surface charge of the nanomaterials and pepsin (free and immobilized) was determined using the zetasizer nano ZS and disposable capillary cell DTS 1061 (Malvern Instruments, Worcestershire, UK). All measurements were performed at room temperature with a refractive index of 2.4 and an equilibration time of 2 min.

### 2.9 Antioxidant Activity of Casein Hydrolysates

The in vitro antioxidant activity of peptides generated from casein hydrolysis was determined based on ABTS (2,2‐azinobis (3‐ethylbenzothiazoline‐6‐sulphonate) diammonium salt), DPPH (2,2‐diphenyl‐1‐picrylhydraxyl), FRAP (reducing power by inhibition of iron ion), and inhibition of β‐carotene acid‐linoleic oxidation. In this stage, casein hydrolysis was performed as previously described to determine the enzymatic activity of immobilized pepsin.

#### 2.9.1 ABTS

Stock solutions of 7 mM ABTS reagent and 140 mM potassium persulfate were prepared first in an aqueous medium to determine the antioxidant activity. To prepare the ABTS^•+^ radical, 5 mL of the ABTS solution was added to 88 µL of the persulfate solution and kept for 16 h in protection from the light. Then, the mixture was diluted in ethyl alcohol until the absorbance reached 0.7 at a wavelength of 734 nm. Note that 15 µL of the sample (casein hydrolysates) was added to 1.5 mL of the ABTS^•+^ solution and kept in the dark for 6 min, and the absorbance was measured at 734 nm. The assay is based on the reduction of the ABTS^•^^+^ radical, which has a characteristic blue‐green color and is relatively stable, by antioxidant compounds, resulting in the formation of the colorless neutral ABTS molecule [31]. The antioxidant activity was expressed as the percentage of ABTS^• +^ radical reduction, as described in Equation (1).

(1) $$\%\;\mathrm{inhibition}=\frac{\mathrm{Ab}s_{\mathrm{ABT}S^{•+}\;}-\mathrm{Ab}s_{\mathrm{sample}}}{\mathrm{Ab}s_{\mathrm{ABT}S^{•+}}}\;\times 100$$

#### 2.9.2 DPPH

The stock solution of DPPH (0.2 mM) was prepared in ethanol, with an initial absorbance of 0.6‐0.7. It was mixed with the hydrolysate solution in a 1:1 ratio and incubated at room temperature in the dark for 30 min. Absorbance was measured at 515 nm. Ethanol was used as the spectrophotometer blank. Antioxidant activity was determined from the percentage of DPPH radical inhibition (%) [32]. (Equation 2).

(2) $$\%\;\mathrm{DPPH}\;\mathrm{inhibition}=\frac{\mathrm{Ab}s_{\mathrm{DPPH}}-\mathrm{Ab}s_{\mathrm{sample}}}{\mathrm{Ab}s_{\mathrm{DPPH}}}\;\times 100$$

#### 2.9.3 FRAP

The FRAP assay as determined as described by Liu et al. [33] with modifications. Initially, a FRAP solution was prepared by combining 10 mM TPTZ (2,4,6‐Tris(2‐pyridyl)‐s‐triazine) in 40 mM HCl, 20 mM ferric chloride hexahydrate, and 0.3 M acetic buffer (pH 3.6) in a 1:1:10 (v/v/v) ratio. Subsequently, 50 µL of the casein hydrolysate sample was mixed with 150 µL of water + 1500 µL of the FRAP solution. The mixture was incubated in a thermostatic bath at 30°C for 30 min, and the absorbance was measured at 595 nm. The blank was prepared under the same conditions, excluding the sample. The calibration curve was prepared with ferrous sulfate, and FRAP values were expressed in terms of µM Fe (II) equivalent per µL of the sample.

#### 2.9.4 Inhibition of β‐Carotene‐Linoleic Acid Oxidation

First, distilled water was saturated with oxygen for 30 min. The rational mixture formed by 40 µL of linoleic acid, 530 µL of Tween 40, 50 µL of the β‐carotene solution (10 mg/mL in chloroform), and 1000 µL of chloroform was kept under stirring at 50°C for approximately 15 min until the solvent was removed. Then, aliquots of saturated water were added to the reaction mixture until an absorbance of 0.6–0.7 at 470 nm was obtained. Note that 5.0 mL of the mixture was added to 400 µL of the hydrolysates, and absorbance was determined before and after incubation at 50°C in a thermostatic bath for 120 min. Water saturated with oxygen was used for the blank. The results were expressed in the percentage of oxidation inhibition (Equation 3). The results are expressed as a percentage of oxidation protection [32].

(3) $$\%\;\mathrm{Protection}=\left[1-\left(\frac{\mathrm{Ab}s_{0\;\mathrm{sample}}-\mathrm{Ab}s_{120\;\mathrm{sample}}}{\mathrm{Ab}s_{0\;\mathrm{control}}-\mathrm{Ab}s_{120\;\mathrm{control}}}\right)\right]\;\times \;100$$

### 2.10 Characterization of Casein Hydrolysates by Reversed‐Phase High‐Performance Liquid Chromatography

The characterization of peptides obtained from the crude extract and immobilized proteases was characterized by reversed‐phase high‐performance liquid chromatography (RP‐HPLC) [34]. A C18 column coupled to a pre‐column connected to a system (HPLC SHIMADZU DGU‐20A5R, equipped with a UV‐DA detector and automatic injection). Two mobile phases were used: the first (A) was formed by a solution of acetonitrile (5.0% v/v) and trifluoroacetic acid (0.1% v/v), and the second phase was formed by acetonitrile (50% v/v) and trifluoroacetic acid (0.1%). Note that 20 µL of the sample was injected into the column at a flow rate of 1.0 mL/min of phases A and B for 70 min. The flow started with 100% phase A and reduced linearly for 60 min until 100% of the flow was formed by B. From 60 to 65 min, a new linear gradient was established to maintain a flow containing 100% A, which was maintained until the end of the analysis. The oven temperature was maintained at 30°C, and the peptides were detected at 215 nm on a UV‐Vis detector.

### 2.11 Statistical Analysis

Statistical differences between the supports studied in terms of immobilization capacity and immobilized pepsin were assessed using the Tukey test (α = 0.05), using the software SAS OnDemand for Academics.

## 3 Results and Discussion

### 3.1 Pepsin Immobilization Tests on GO and RGO

The effect of pepsin concentration on GO adsorptive capacity, immobilization efficiency, and biocatalyst activity is presented in Table 1. These measurements provided, respectively, the amount of enzyme adsorbed per gram of support, the percentage of the initial enzyme immobilized on the support relative to the total amount of enzyme added to the system, and the proteolytic activity exhibited by the enzyme after the immobilization process. The highest GO adsorptive capacity was observed at the highest pepsin concentrations, indicating that a greater amount of enzyme was immobilized per support mass, reaching 1.4073 mg of enzyme/mg of GO in the assay performed with 10 mg/mL of pepsin.

**TABLE 1:** Effect of pepsin concentration on the absorptive capacity and immobilization efficiency of GO, and activity recovery of immobilized pepsin.

Pepsin concentration (mg/mL)	Adsorptive capacity (mg of enzyme/mg of GO)	Immobilization efficiency (%)	Activity recovery (%)
2.5	0.386 ± 0.024	92.719 ± 0.080	5.284 ± 0.002
5.0	0.719 ± 0.155	87.441 ± 0.005	6.590 ± 0.030
7.5	1.065 ± 0.037	85.229 ± 0.040	6.376 ± 0.020
10.0	1.407 ± 0.048	84.441 ± 0.056	6.572 ± 0.050

*Note*: Values are expressed as mean ± SD (*N* = 3).

Although the total amount of pepsin adsorbed per unit mass of the support increased with increasing concentration, a reduction in immobilization efficiency was observed for higher pepsin concentrations. At 2.5 mg/mL, the efficiency reached its maximum, approximately 92%, while at 10 mg/mL, it decreased to 84.4%. The decrease in the values obtained for this parameter indicates that excess enzyme remained in solution or was weakly bound to available sites on the GO surface and was desorbed during the washing steps.

The high content of immobilized pepsin in GO may be due to the coexistence of multiple interactions between the enzyme and GO, including electrostatic, hydrophobic, and π–π interactions. Ionic interactions, for example, are favored by the net negative charge of GO in a wide pH range and by the net positive charge of pepsin under immobilization conditions. In addition, aromatic amino acid residues allow π–π interactions with the surface of the support [35]. However, increasing the concentration of pepsin can also lead to saturation of available binding sites, thus limiting the efficient immobilization of additional enzyme molecules [20].

Another important aspect of pepsin immobilization on GO is the decreased enzyme activity compared to other supports [8, 36]. After immobilization, pepsin maintained only 5.28%–6.38% of the activity of its free form. When evaluating the effect of pepsin concentration, Duan and Cheng [13] proposed that the reduction in enzymatic activity at high concentrations may be associated with steric hindrance since the excess enzyme may have obstructed or even destroyed (due to autohydrolysis) the enzyme's binding sites to the substrate.

To minimize the steric effects associated with the reduction of enzymatic activity, a lower concentration of pepsin was used in further assays, enhancing substrate accessibility and reducing autodigestion. We also tested RGO as an immobilization support due to its different surface properties compared to GO. As exhibited in Table 2, it was not possible to determine the proteolytic activity of Pepsin‐GO and Pepsin‐RGO biocatalysts. GO maintained its efficiency in enzyme immobilization at about 90%, while the RGO efficiency decreased to 80%. The interaction between the hydrophobic groups of the enzyme and RGO can affect the stability and, consequently, the proteolysis, as these groups play an essential role in enzyme stabilization.

**TABLE 2:** Immobilization efficiency of graphene oxide (GO), reduced graphene oxide (RGO), and the enzymatic activity of immobilized pepsin.

Parameters	Pepsin	Pepsin‐GO	Pepsin‐RGO
Immobilization efficiency (%)	—	90.000 ± 1.570	85.000 ± 0.109
Proteolytic activity (U)	0.050 ± 0.054	Nd	Nd

*Note*: Enzymatic activity was not observed for the immobilized enzyme. Values are expressed as mean ± SD (*N* = 3).

Abbreviation: Nd: not detectable.

### 3.2 Pepsin Immobilization on Different Nanomaterials

Pepsin immobilization was also assessed using different nanomaterials, such as boron nitride (BN) nanosheets and BN functionalized with NH_2_, gold nanoparticles (GNP) of 13 and 45 nm, and GNP functionalized with cysteine (Table 3). For all materials evaluated, there was no significant difference in immobilization efficiency.

**TABLE 3:** Immobilization efficiency of the nanomaterials and proteolytic activity of free and immobilized pepsin on BN and GNP.

Sample	Immobilization efficiency (%)	Proteolytic activity (U)
Free pepsin	—	0.050b ± 0.054
BN	99.000a ± 0.002	0.094a ± 0.024
BN‐NH_2_	99.870a ± 0.004	0.098a ± 0.015
GNP 13 nm	99.043a ± 0.030	0.043b ± 0.008
GNP 45 nm	99.143a ± 0.090	0.104^a^ ± 0.015
GNP/Cys 13 nm	99.097a ± 0.100	0.052b ± 0.004
GNP/Cys 45 nm	99.543a ± 0.053	0.053b ± 0.006

*Note*: Mean values followed by the same letter do not differ in the same column (*p* < 0.05). Values are expressed as mean ± SD (*N* = 3).

The high binding capacity of pepsin to the BN can be attributed to the support's high surface area, approximately 2600 m^2^/g, as demonstrated by Gao et al. [37]. The presence of the amino group in BN‐NH_2_ provides additional binding sites with the enzyme, as demonstrated by Fernando et al. [25], resulting in almost all the enzyme content being immobilized.

Regarding the immobilization of pepsin in GNP, the particle diameter did not influence the binding capacity of pepsin in the presence of ligands (cysteine) on the surface of the nanomaterial. In all conditions, GNP presented a binding capacity of approximately 100%. The high enzyme loading of pepsin to GNP may be associated with the relatively small dynamic diameter of pepsin, approximately 3.9 nm, compared to the size of the nanoparticles used in the present study, thus forming a favorable microenvironment for the immobilization of this enzyme on the surface of the material [38]. Furthermore, for GNP/Cys, the functionalization allows more significant interaction between the thiol group of cysteine and the carboxyl and amino groups of pepsin, resulting in a high content of immobilized pepsin [39].

Proteolytic activity is influenced by conformational changes resulting from enzyme–support interactions or the enzyme's orientation after immobilization, which can either hinder or enhance substrate access to the active site [17]. Immobilization did not significantly reduce proteolytic activity, except when 13 nm GNP was used as a support. The supports BN, BN‐NH_2_, and GNP 45 nm led to an increase in activity, possibly because of the pepsin's orientation after immobilization, where the active site is directed away from the nanoparticle surface, enhancing the accessibility to the substrate [40]. GNP functionalization with cysteine contributed to maintaining the hydrolysis capacity of the biocatalysts. There was no significant difference between the activity of the Pepsin‐BN and Pepsin‐BN‐NH_2_ biocatalysts, which were approximately 1.9 times higher than that of the free enzyme, which corroborates previous studies that used BN for enzyme immobilization [41].

The increase in the GNP particle diameter from 13 to 45 nm leads to a rise in the enzymatic activity of immobilized pepsin from 0.043 to 0.104 U. As per the studies by Vertegel et al. [42], the smaller the particle size and the greater its curvature, the fewer the interaction sites between the enzyme and the nanoparticle. As a result, there were fewer disturbances in the enzyme conformation and in proteolytic activity. Although significant changes in conformation can lead to a reduction in enzymatic activity, either because the enzyme becomes more rigid or due to the loss of stability caused by interactions with the support, it seems that the changes induced by immobilization in GNP45 nm may have instead enhanced enzymatic activity [22].

### 3.3 Characterization of Nanoparticles and Immobilized Pepsin

The zeta potential values ​​for the enzyme, the supports, and the resulting biocatalysts (Table 4) showed that all samples exhibited a negative surface charge. Given that both the enzyme and the supports were negatively charged under the immobilization conditions, electrostatic interactions could not be considered the primary driving force. The immobilization on GNP is probably a result of non‐electrostatic interactions, such as hydrophobic interactions or van der Waals forces, that characterize physical adsorption. For the support GNP/Cys, the thiol group of cysteine may promote a more specific interaction with pepsin, potentially forming covalent bonds with the carboxyl group of the enzyme [43].

**TABLE 4:** Zeta potential (mV) of free and immobilized pepsin on nanomaterials.

Sample	Zeta potential (mV)
Pepsin	−10.6 ± 11.5
Gold nanoparticle	
GNP 13 nm	−32.0 ± 16.1
GNP 45 nm	−36.7 ± 11.6
Pepsin‐GNP 13 nm	−30.5 ± 13.9
Pepsin‐GNP 45 nm	−27.6 ± 19.9
GNP/Cys 13 nm	−20.7 ± 8.9
GNP/Cys 45 nm	−22.87 ± 14.9
Pepsin‐GNP/Cys 13 nm	−18.6 ± 14.5
Pepsin‐GNP/Cys 45 nm	−17.3 ± 11.7
Boron nitride nanosheets	
BN	−37.1 ± 15.0
BN‐NH_2_	−37.3 ± 12.6
Pepsin‐BN	−22.8 ± 7.58
Pepsin‐BN‐NH_2_	−27.3 ± 9.55

*Note*: Values are expressed as mean ± SD (*N* = 3).

The viability of cysteine as a surface functionalizing agent for the immobilization of proteins and enzymes in gold nanoparticles is widely documented in the literature [17, 38, 44]. The magnitude of the zeta potential can also indicate information about the stability of the particles. The greater the magnitude, as observed in this study, the stronger the electrostatic repulsion and the lower the chance of the particles agglomerating, thus maintaining their stability [38].

Interactions between enzymes and nanomaterials can be studied using UV‐visible spectroscopy. This technique is possible because aromatic amino acids such as phenylalanine, tryptophan, and tyrosine absorb light in the UV region. Therefore, interactions between these amino acids and other compounds can generate spectra with different characteristics. The spectrum generated by GO presents a maximum peak at 230 nm attributed to π‐π interactions (Figure 1).

**FIGURE 1:**
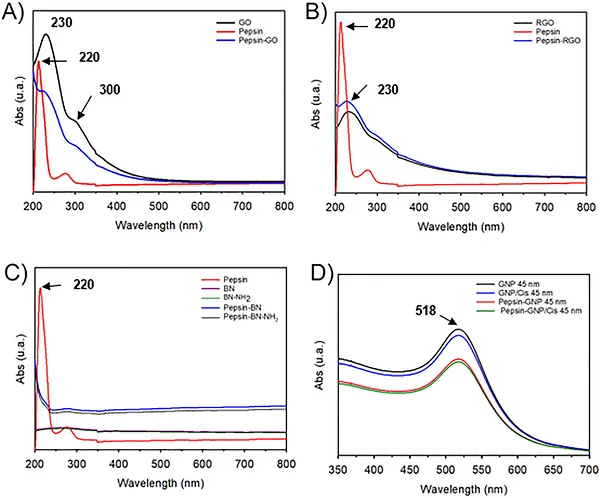
The UV‐visible spectrum of pepsin is free and immobilized on nanomaterials. (A) GO, (B) RGO, (C) BN and BN‐NH_2_, and (D) GNP and GNP/Cys.

For RGO, there was a reduction in the intensity of this peak, indicating that the reduction using L‐ascorbic acid effectively occurred. A change in the characteristic spectrum of GO was observed around 230 nm after the immobilization of pepsin due to the interaction between the support and the enzyme amino acids, indicating the formation of the pepsin‐GO biocatalyst. The BN and BN‐NH_2_ nanosheets did not present an absorption peak. Only in the pepsin‐BN and pepsin‐BN‐NH_2_ samples was an increase in absorbance at a wavelength below 250 nm, which also occurs due to pepsin amino acids. This behavior reflects the effective binding of the enzyme to BN and BN‐NH_2_ [15, 18]. Unlike the other supports studied, the main change in the GNP spectra after pepsin immobilization is the change in absorbance peaks, which can also be observed after GNP functionalization.

### 3.4 Changes in Pepsin Conformation After Immobilization

To evaluate the changes in the secondary structure of pepsin after immobilization in nanomaterials, circular dichroism spectra of the immobilized enzyme and its free form were obtained (Figure 2). CD spectroscopy is a sensitive and efficient method widely used in studies aimed at elucidating changes in protein structure. Pepsin exhibits a minimum peak at 213 nm, indicating the predominant presence of the β‐sheet structure [11]. There was no significant modification in the characteristic profile of the spectra of the immobilized enzyme. However, a reduction in molar negative ellipticity was observed. For the biocatalysts Pepsin‐BN‐NH_2_‐ and Pepsin‐GNP/Cys 45, a blue shift of the maximum peak can be noted. These results suggest that immobilization resulted in changes in the secondary structure of pepsin.

**FIGURE 2:**
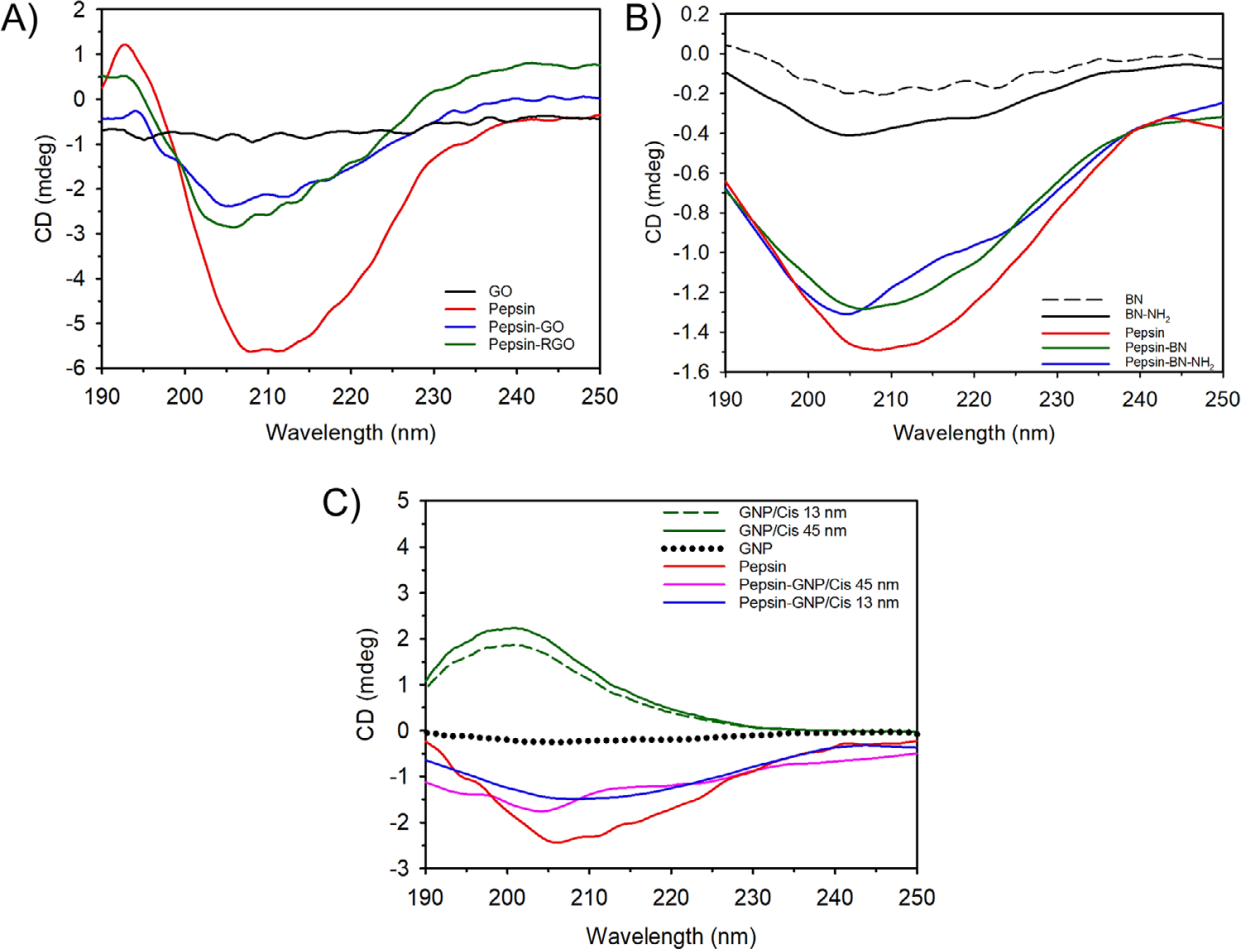
CD spectra of pepsin immobilized on GO and RGO (A), BN and BN‐NH_2_ (B), and GNP and GNP/Cys (C).

To better evaluate changes in pepsin's secondary structure after immobilization, the α‐helix, β‐sheet, β‐turn, and random coil fractions were calculated using the BeStSel online server. Results are presented in Table 5.

**TABLE 5:** Secondary structure analysis from free and immobilized pepsin.

Sample	α‐Helix (%)	β‐Sheet (%)	β‐Turn (%)	Random coil (%)
Free pepsin	8.4	22.9	13.8	51.1
Pepsin‐GO	6.2	27.9	11.1	54.8
Pepsin‐RGO	2.5	41.0	15.4	41.1
Pepsin‐GNP13	6.7	21.6	16.4	55.3
Pepsin‐GNP45	6.1	25.4	14.7	53.8
Pepsin‐GNP/Cys 13	2.4	34.4	16.2	47.0
Pepsin‐GNP/Cys 45	2.2	32.7	17.9	47.9
Pepsin‐BN	8.2	22.6	15.4	53.7
Pepsin‐BN‐NH_2_	4.7	23.0	17.1	55.2

The use of GNP as support resulted in a reduction in α‐helix fractions, approximately 20% for GNP13 and 27.3% for GNP45. Meanwhile, functionalization of GNP with cysteine resulted in a significant change in the α‐helix content, with a reduction of about 71%. No significant variations in this conformation were observed between different GNP sizes within the functionalized and non‐functionalized groups.

There was a reduction to 44% in the α‐helix content of pepsin immobilized on BN‐NH_2_, indicating the interaction between NH_2_ and the enzyme. The use of RGO led to a reduction of approximately 99% in α‐helix content, suggesting the loss of its three‐dimensional conformation and of its catalytic activity, which corroborates the results presented for the proteolytic activity of these biocatalysts (Section 3.1).

Regarding the β‐sheet fractions, the content found in Pepsin‐GNP‐Cys 45 was about 43% higher compared to free pepsin. For the Pepsin‐BN, the fraction of this conformation was maintained. Similar to the α‐helix structure, immobilization on functionalized GNPs led to more pronounced changes than those caused by non‐functionalized GNPs, with a 44% increase in the β‐sheet content of pepsin immobilized on GNP/Cys 45 nm compared with free pepsin. These conformational changes can be associated with enzyme–support interactions, which may alter the exposure and orientation of the catalytic site and influence the dynamics of the catalytic activity [11, 45].

### 3.5 Determination of the Antioxidant Activity of Casein Hydrolysates

Considering that higher enzymatic activity may lead to greater peptide generation, the biocatalysts with the highest catalytic activity were selected for the production of casein hydrolysates (Pepsin‐GNP 45 nm, Pepsin‐BN, and Pepsin‐BN‐NH_2_) (Table 6).

**TABLE 6:** Antioxidant properties of bovine casein hydrolysates obtained from pepsin in its free and immobilized form.

	Antioxidant activity			
	DPPH (% of inhibition)	ABTS (% of inhibition)	FRAP (µM/µL)	β‐Carotene/linoleic acid (% protection)
Pepsin	67.440a ± 0.560	51.310a ± 1.670	241.000a ± 7.070	62.530a ± 2.980
Pepsin‐GNP 45 nm	62.010b ± 0.470	29.060b ± 0.790	231.000a ± 1.410	64.630a ± 5.550
Pepsin‐BN	65.310a ± 2.450	29.190b ± 0.970	205.100a ± 21.210	56.420b ± 1.490
Pepsin‐BN‐NH_2_	68.110a ± 1.510	30.940b ± 2.210	175.000b ± 7.070	65.050a ± 6.550

*Note*: Mean values followed by the same letter do not differ in the same column (*p* < 0.05). Values are expressed as mean ± SD (*N* = 3).

The peptides derived from the Pepsin‐GNP 45 nm biocatalyst exhibited the lowest capacity to inhibit the oxidation of DPPH and ABTS radicals. For ABTS radical inhibition, peptides obtained from free pepsin showed better results (51.31%) compared with those from the immobilized enzyme (29.06%–30.94%). Hydrolysates obtained from Pepsin‐BN and Pepsin‐BN‐NH_2_ showed a lower capacity to inhibit β‐carotene oxidation and reduce ferric ions (Fe^3^^+^).

The reduction in antioxidant activity can be due to alterations in the peptide profile obtained after pepsin immobilization. These changes probably result from conformational modifications in the enzyme that affect its specificity. Although no significant increase in antioxidant activity was observed, the results are promising, as they demonstrate that immobilization can modulate the type of peptide produced and may be strategically explored depending on the target application, such as antihypertensive, antimicrobial, or functional food properties.

### 3.6 Characterization of Casein Hydrolysates by RP‐HPLC

In this study, RP‐HPLC was employed to compare the chromatographic profiles of the protein hydrolysates. This technique separates the hydrolysates based on their hydrophobicity, providing information on changes in peptide distribution resulting from the action of the immobilized enzyme [34].

Figure 3 shows the chromatograms of the hydrolysates. After around 10 min, the presence of peaks with higher frequency indicates the action of free and immobilized pepsin on the casein molecule and its conversion to low molecular weight peptides. The number of peaks of the hydrolysates obtained from free enzyme in the time range of 20–50 min indicates more activity in casein digestion, compared with the immobilized pepsin at the same time. This difference in the peptide profile and concentration can be linked to a reduction in the inhibition of ABTS radical oxidation by peptides obtained from immobilized pepsin compared to those from free pepsin form.

**FIGURE 3:**
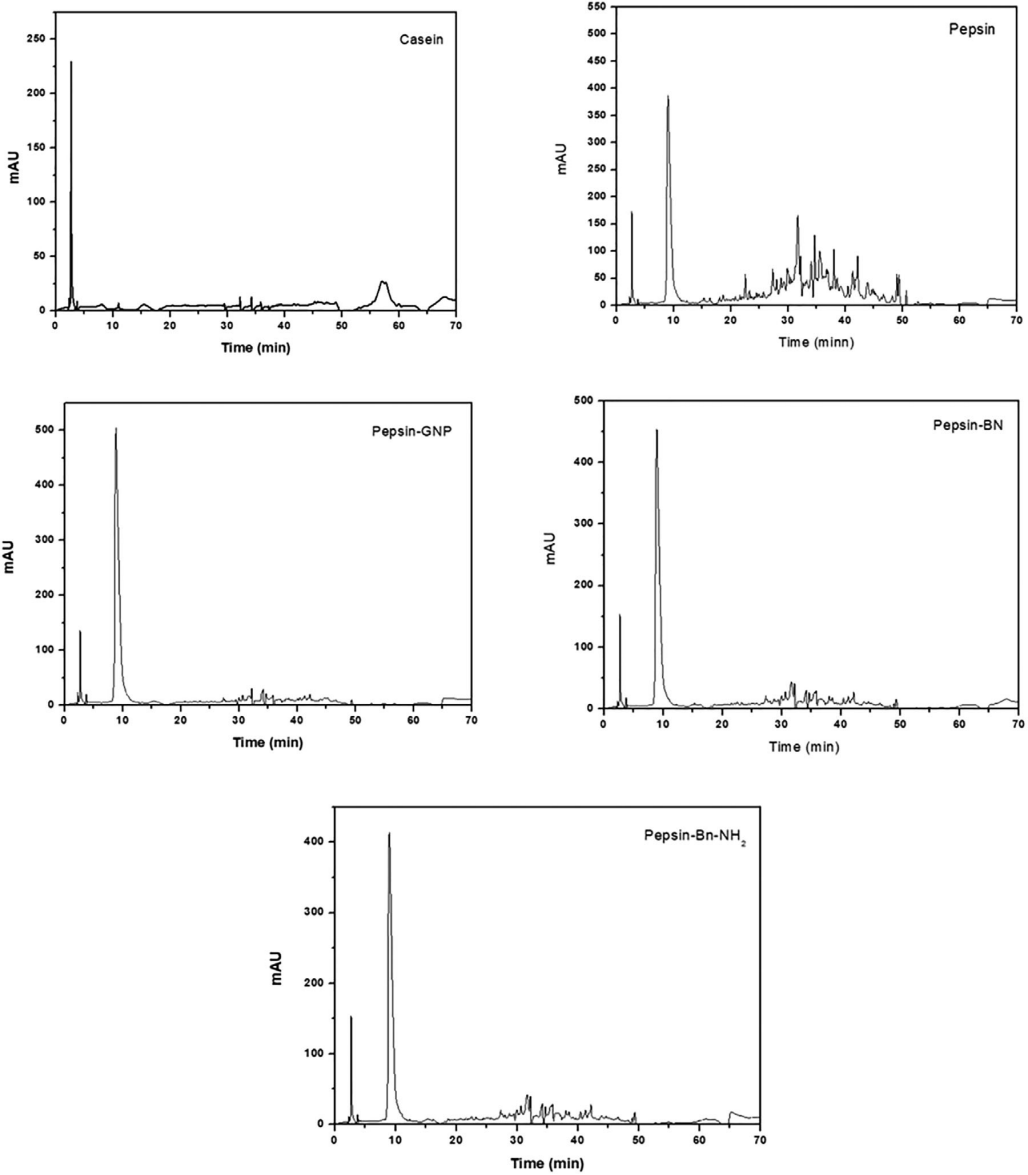
Chromatograms of bovine casein hydrolysates obtained from free and immobilized pepsin.

Such differences are a result of changes in the secondary structure of pepsin, as indicated by the CD spectrum (Section 3.4), which possibly caused changes in the dynamics of the pepsin–substrate interaction. The chromatograms indicate similar peptide distribution patterns for the hydrolysates generated by Pepsin‐BN, Pepsin‐BN‐NH_2_, and Pepsin‐GNP.

## 4 Conclusions

The results of this study highlight how the type of nanomaterial used as a support can influence the immobilization of pepsin and its subsequent functional activity. GNPs, particularly those with a diameter of 45 nm, exhibited higher immobilization efficiency and an increase in enzymatic activity compared to the free enzyme. However, the protein hydrolysates obtained using the enzyme immobilized on this support did not show significant differences in antioxidant activity, as assessed by the FRAP method and the β‐carotene/linoleic acid complex protection assay, when compared to the free enzyme. Overall, none of the nanomaterials studied proved consistently effective for producing biopeptides with superior antioxidant properties, with variations depending on the evaluation method used.

These differences observed may be related to changes in the three‐dimensional conformation of pepsin caused by the immobilization process, which affects its catalytic specificity and, consequently, modifies the profile of the peptides generated. The choice of the ideal support should consider not only the immobilization efficiency and stability of the enzyme, but also the effects on the functionality of the hydrolysates generated. Despite this, the results observed are promising, since enzyme immobilization presents operational advantages, such as reuse of the enzyme, increased stability, and application as biocatalysts in the production of compounds of importance to the food and pharmaceutical industries.

Furthermore, it is expected that the findings of this work will encourage further studies on the use of these nanomaterials, particularly graphene oxide and its derivatives, to better understand the conformational changes induced in the enzyme after immobilization and their effects on catalytic activity. Such investigations are essential for the development of more effective nanobiocatalysts for industrial applications.

## Author Contributions

**Annie N. Alves**: conceptualization, investigation, writing – original draft, methodology, validation, visualization, writing – review and editing, data curation. **Márjorie C. P. Porfirio**: writing – review and editing, writing – original draft, methodology, validation, data curation. **Priscilla A. Nascimento**: conceptualization, investigation, methodology. **Jéssica F. Borges**: investigation, methodology. **Wenrong Yang**: conceptualization, visualization, project administration, supervision, data curation, resources. **Motilal Mathesh**: supervision, visualization, project administration, methodology, data curation. **Paulo Bonomo**: supervision, data curation, resources. **Rafael da C. I. Fontan**: funding acquisition, resources. **Renata C. F. Bonomo**: conceptualization, investigation, funding acquisition, resources, supervision, data curation, project administration, writing – original draft, writing – review and editing, visualization, methodology.

## Funding

This study was supported by Universidade Estadual do Sudoeste da Bahia.

## Conflicts of Interest

The authors declare no conflicts of interest.

## Data Availability

The authors have nothing to report.
